# Radio frequency electromagnetic radiations interfere with the Leydig cell functions *in-vitro*

**DOI:** 10.1371/journal.pone.0299017

**Published:** 2024-05-17

**Authors:** Pooja Jangid, Umesh Rai, Rajeev Singh

**Affiliations:** 1 Department of Environmental Studies, Satyawati College, University of Delhi, Delhi, India; 2 Department of Zoology, University of Delhi, Delhi, India; Dr. Anjali Chatterji Regional Research Institute for Homeopathy, INDIA

## Abstract

A growing threat to male infertility has become a major concern for the human population due to the advent of modern technologies as a source of radiofrequency radiation (RFR). Since these technologies have become an integral part of our daily lives, thus, it becomes necessary to know the impression of such radiations on human health. In view of this, the current study aims to focus on the biological effects of radiofrequency electromagnetic radiations on mouse Leydig cell line (TM3) in a time-dependent manner. TM3 cells were exposed to RFR emitted from 4G cell phone and also exposed to a particular frequency of 1800 MHz and 2450 MHz from RFR exposure system. The cells were then evaluated for different parameters such as cell viability, cell proliferation, testosterone production, and ROS generation. A considerable reduction in the testosterone levels and proliferation rate of TM3 cells were observed at 120 min of exposure as compared to the control group in all exposure settings. Conversely, the intracellular ROS levels showed a significant rise at 60, 90 and 120 min of exposure in both mobile phone and 2450 MHz exposure groups. However, RFR treatment for different time durations (15, 30, 45, 60, 90, and 120 min) did not have significant effect on cell viability at any of the exposure condition (2450 MHz, 1800 MHz, and mobile phone radiation). Therefore, our findings concluded with the negative impact of radiofrequency electromagnetic radiations on Leydig cell’s physiological functions, which could be a serious concern for male infertility. However, additional studies are required to determine the specific mechanism of RFR action as well as its long-term consequences.

## 1. Introduction

The usage of mobile phones, Wi-Fi, microwave ovens, bluetooth devices etc., has increased in recent years, raising the concerns about their possible health impacts. Most of these devices operate at a standard frequency range of 1800 MHz to 2450 MHz. The radiations emitted from these devices has dramatically increased Radiofrequency radiation (RFR) exposure in everyday life. Specific absorption rate (SAR) measurement is used to assess the impact of RFR on human body. The SAR is a standard unit of measurement for the rate of energy absorption per unit mass of the body when exposed to RFR, based on intensity, exposure period, frequency, polarization, and location of the electromagnetic device. If the electromagnetic device is close by, the absorption rate will be greater [1]. Radiofrequency radiation has been categorized as a "possible carcinogen" (category 2B) to humans by the International Agency for Research on Cancer [2]. With the rise in popularity of Wi-Fi, wireless communication and bluetooth devices etc., there is a fear that exposure to RFR released by these devices may cause health problems, such as sleep difficulties, an increased risk of cancer and immunological disorders, and might have a negative impact on both male and female fertility [3–7].

Male fertility has been found to be significantly affected in recent years and various studies have connected Wi-Fi and mobile phone usage to negative effects on a male’s reproductive system [8–11]. According to a study, exposure of ejaculated semen to RFR from Wi-Fi devices resulted insignificantly altered sperm motility and morphology [11]. Since men typically carry their mobile phones in their pockets or in close proximity to their reproductive organs and the new practice of using bluetooth or wireless earphones while talking, has increased the exposure period of the reproductive organs to radiation. Also, when using a laptop for work, most individuals place it on their laps, a few centimetres from their gonads, to access the internet broadcast signal or Wi-Fi which increases the gonads exposure to RFR. Therefore, it is crucial to evaluate the impact of RFR on male fertility.

Numerous studies on the effects of radiofrequency electromagnetic radiation (RF-EMR) on male reproduction have been published recently, with contradictory outcomes [5, 12–15]. Some of them suggested adverse outcomes, whereas other studies showed negligible effects. Also, the majority of RF-EMR research on testis function has been conducted *in vivo* to assess the radiation’s overall effects on fertility or testis function. However, the studies have not provided any information on the radiation’s effects when RFR is given *in vitro*. In this context, we examined the time dependent effects of RF-EMR (1800 MHz, 2450 MHz, and mobile phone radiation) on the mouse Leydig cell line (TM3). The essential sex hormone in men is testosterone, which is secreted by Leydig cells and necessary for maintaining reproductive functions [16, 17]. Leydig cells secretory activity was discovered to be impaired by an Electromagnetic radiation (EMR) of 220 MHz [18]. A new study on mouse spermatozoa revealed that complex III of the respiratory chain in mitochondria is the primary target of radiofrequency radiation, which, after inflicting oxidative DNA damage, eventually results in a reduction in sperm motility [19]. Other aspects of cellular function, however, were not examined in this study. In this connection, our study aims to determine how RF-EMR (2450 MHz, 1800 MHz, and mobile phone radiation) affects the viability, proliferation, testosterone secretion, and intracellular ROS generation in Leydig cells (TM-3).

## 2. Materials and methods

### 2.1 Cell culture

Mouse Leydig (TM-3) cells obtained from the American Type Culture Collection (ATCC®, USA) were cultured using a standard sterile cell culture technique. Medium consisted of Dulbecco’s Modified Eagle’s Medium F12 (DMEM/F12, 1:1 Mixture, Himedia) with 10% Fetal Bovine Serum (FBS; Gibco TM) and 1% Penicillin-Streptomycin (Sigma). The cells were maintained at 37°C in humidified air containing 5% CO_2_. Routinely, TM-3 cells were cultured in T-75 culture flasks containing 15–18 ml complete cell culture medium. They were sub-cultured, when 80–90% confluent, cells were harvested by using 0.25% Trypsin-EDTA 1X Solution (Himedia) and were used to conduct experiments.

### 2.2 Reagents and culture media

Reagent-grade chemicals were mostly obtained from Sigma and Himedia. Heat-inactivated fetal bovine serum (FBS, Himedia), Dulbecco’s Modified Eagle’s Medium F12/ (DMEM/F12, Himedia). 1% Penicillin-Streptomycin (Sigma), MTT [3-(4,5-dimethyl thiazolyl-2)-2,5-diphenyl tetrazolium bromide] were procured from Sigma-Aldrich Chemicals (St. Louis, MO). 0.25% Trypsin-EDTA 1X Solution (Himedia), DCFH-DA (Sigma) and other routine chemicals were purchased from Sigma/Merck.

### 2.3. Cell exposure

#### 2.3.1 Exposure using mobile phone

RFR exposure and Sham exposure were performed as described earlier by Yadav et al. 2023 [20]. Briefly, a 4G mobile phone (a Redmi Note 7) was used for exposure at varying time durations at a power density of 0.224 W/m^2^ in incubator at 37°C with 5% CO_2_. Cells were exposed in 15 mL round-bottom tubes at a concentration of 1 × 10^6^ cells/mL in each tube. As per the manufacturer’s claim, the mobile phone has a SAR of 0.962 W/kg (head) and 0.838 W/kg (body) (distance 15 mm). Calibration was accomplished by measuring physical parameters of the cell-phone with the Narda system 520. Herein, during the exposure, no temperature change was observed. Since the background conditions or fields in our experiment were identical for both the sham exposure and the real exposure, the differences that were observed cannot be attributed to these conditions.

#### 2.3.2 Exposure using signal generator

The exposure setup was constituted with a RF signal generator, handheld power meter, a horn antenna, and an incubator/ irradiation chamber. An electromagnetic wave of 1800 MHz and 2450 MHz was generated by a signal generator (Keysights, USA) and emitted by the antenna. TM3 cells in the exponentially growing phase were used for experiments. The temperature difference between the exposure groups and control group did not exceed 0.1°C. The temperature and CO_2_remained constant within the incubator at 37°Cand 5%, respectively, during the exposure period. Cells were exposed in 35 mm Petri dishes at the density of 1 × 10^6^cells/mL and placed 2 cm apart from the horn antenna.

### 2.4. Experimental design

The exponentially growing mouse Leydig cell (TM-3) were treated concurrently as follows:

1. Mobile phone exposure
  - Control Group: Cell cultures were not irradiated.
  - Exposure Group: Cell cultures were exposed to radio frequency electromagnetic radiations emitted from a commercially available 4G mobile phone at talk mode.
2. 2450 MHz exposure
  - Control Group: Cell cultures were not irradiated with any radiation.
  - Exposure Group: Cell cultures were irradiated in an exposure system with a frequency set at 2450 MHz.
3. 1800 MHz exposure
  - Control Group: Cell cultures were not irradiated with any radiation.
  - 1800 MHz radiation exposure: Cell cultures were irradiated in an exposure system with a frequency set at 1800 MHz.

All the experiments were carried out at 15, 30, 45, 60, 90, and 120 min of exposure durations. After irradiation, the cells were processed further for different assays.

### 2.5. *In vitro* experiments

#### 2.5.1 Cell viability assay

The viability of Leydig cells was assessed *via* trypan blue dye exclusion method [21]. Once trypsinized, 10 μl of TM3 cell suspension (~ 1× 10^5^) was incubated for 3 min at 37°C with 1:1 dilution of 0.4% trypan blue dye (Sigma). 10 μl of dye treated cell suspension were further placed in the hemocytometer and observed under microscope. The number of viable and damaged cells were represented by the number of unstained and stained cells, respectively. The percentage of viable cells was calculated after counting the total number of cells.

#### 2.5.2 Cell proliferation assay

The cell proliferation assay was performed using MTT [22]. After irradiation, 100 μl of TM3 cells at a density of 1x10^5^/well were seeded and cultured in a 96- well plate for 24 hr in CO_2_ incubator at 37°C. After incubation, the media was replaced with MTT (0.5 mg/ml) (Sigma) and incubated further for 3 hr. MTT solution was aspirated once the incubation period was completed, and 250 μl of isopropanol was added to dissolve the purple formazen crystals, formed as a compound of this reaction. Finally, optical density was taken at 450 nm using an ELISA reader (iMark, Bio-Rad).

#### 2.5.3 Testosterone content assay

After irradiation, TM3 cells (1x10^6^ cells/ml) were seeded in a 24-well culture plate and incubated for 24 hr. Following incubation, cells were scraped and culture media was collected in the sterilized eppendorf tubes. Cells were centrifuged at 12000 rpm for 10 min at 4°C and supernatant was collected and stored at -20°C, until the testosterone evaluation was done.

Analysis was further performed using the Testosterone ELISA Kit (Cayman, Item No.: 582701), following the manufacturer’s direction. In brief, 50 μl of testosterone supernatant, 50 μl of the testosterone AChE tracer, and 50 μl of testosterone antiserum were added to each ELISA coated well. Further, after 2 hrof incubation, 5 times washing with the ELISA wash buffer was performed. 200 μl of Ellman’s reagent was added to each well and incubated for 45 min at room temperature in dark. Testosterone concentrations were determined at an optical density of 415nm by using an iMark Bio-Rad ELISA microplate reader.

#### 2.5.4 Intracellular ROS analysis

Intracellular ROS was detected in the exposed and non-exposed groups using 2′, 7′-dichloro-dihydro-fluorescein diacetate (DCFH-DA)(Sigma) staining method. After irradiation, cells (1x10^5^ cells/ml) were processed in 96 black well plate (Corning Incorporated, costar) to analyze intracellular reactive oxygen species and incubated for 24 hours [23]. After 24 hr of incubation, the media was carefully removed and the adherent cells were washed twice with PBS. PBS was replaced with 100 μl DCFH-DA staining solution (10 μM) and incubated further for 45 min at 37°C in the CO_2_ incubator. Once stained, the DCFH-DA solution was removed and the cells were washed twice with PBS. The fluorescence intensity was finally measured using a microplate reader (BioTek, Synergy H1) at an excitation wavelength of 485 nm and emission at 530 nm.

### 2.6 Statistics

All the experiments were repeated at least thrice on independent samples. Experiments were performed in triplicates for each of the parameters studied and the data obtained from an individual experiment (n = 3) was used for statistical analysis. Prism 8 (GraphPad, USA) and Microsoft Excel (2013) were used for statistical analysis. A one-way analysis of variance (ANOVA) with Dunnett’s multiple comparisons test was used to evaluate the statistical significance. The value represents the mean ± SEM of an independent experiment (n = 3 for each assay); differences were estimated statistically significant at a value of p< 0.05. The level of significance was set at **** (p < 0.0001), *** (p < 0.001),** (p< 0.01), and * (p < 0.05).

## 3. Results

### 3.1 Effect of RFR on cell viability

**Fig 1:**
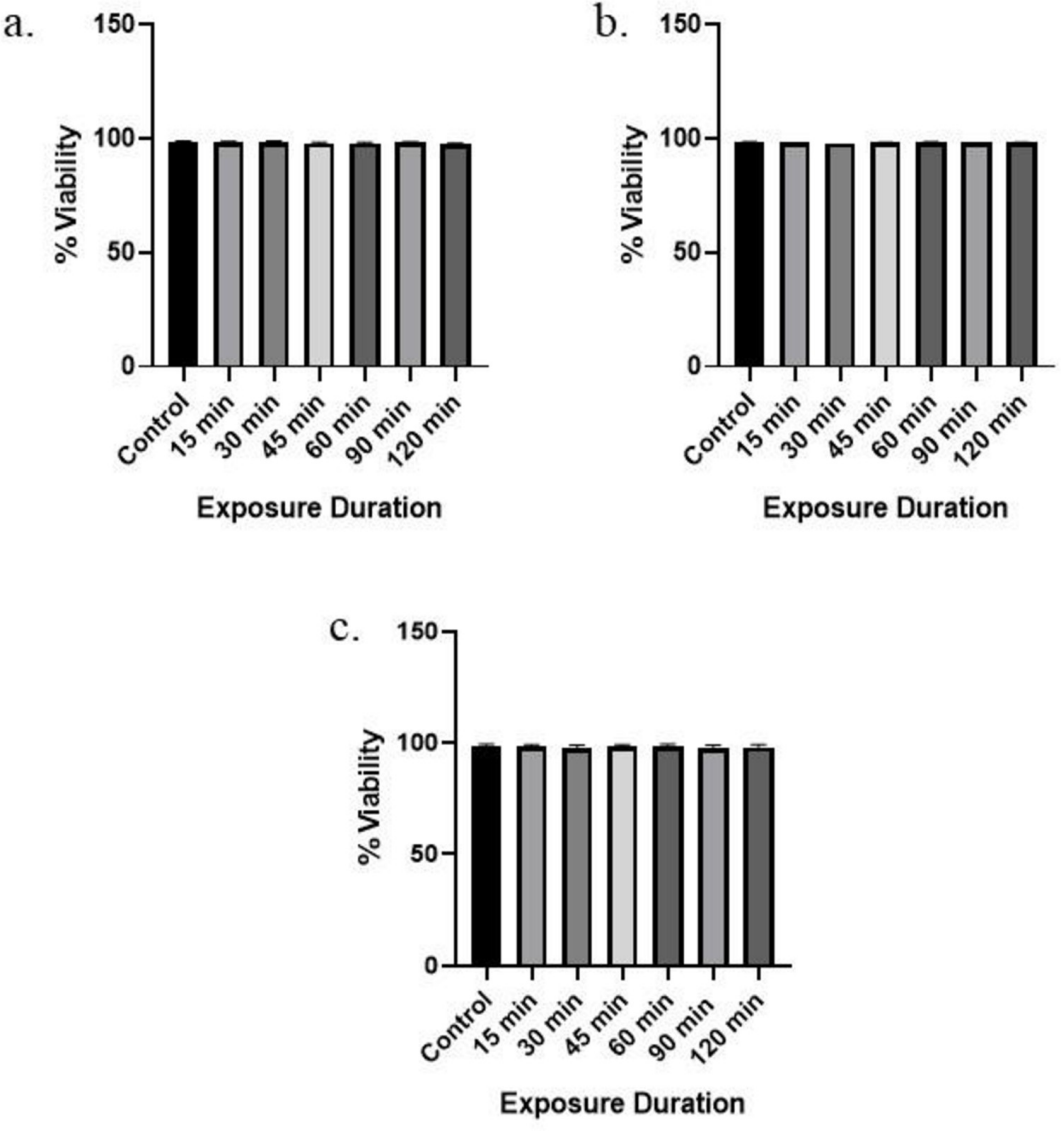
Time-dependent effect of radiation on the viability of mouse Leydig cells. a) Mobile phone radiation, b) 2450 MHz, and c) 1800 MHz. Value represents the mean of one independent experiment (n = 3for each group). Error bars indicate the standard error of mean (SEM) within each group. p<0.05 was considered statistically significant with respect to the control group.

Trypanblue exclusion assay was used to evaluate the cytotoxic effect of RFR (1800 MHz, 2450 MHz, and mobile phone radiation). The Leydig cells were processed immediately after exposure, and the results are presented in Fig 1. According to the trypan blue assay, the viability of cells at all exposure durations was recorded to be approximately 98% of control group in all cases of 1800 MHz (Fig 1C), 2450 MHz (Fig 1B), and mobile phone radiation (Fig 1A). The data showed no significant difference between the irradiated cells’ viability at different time durations (15, 30, 45, 60, 90, and 120 min) and control cells (p > 0.05). Hence, it can be concluded that the 1800 MHz, 2450 MHz, and mobile phone radiation did not affect the cell viability.

### 3.2 Effect of RFR on cell proliferation

**Fig 2:**
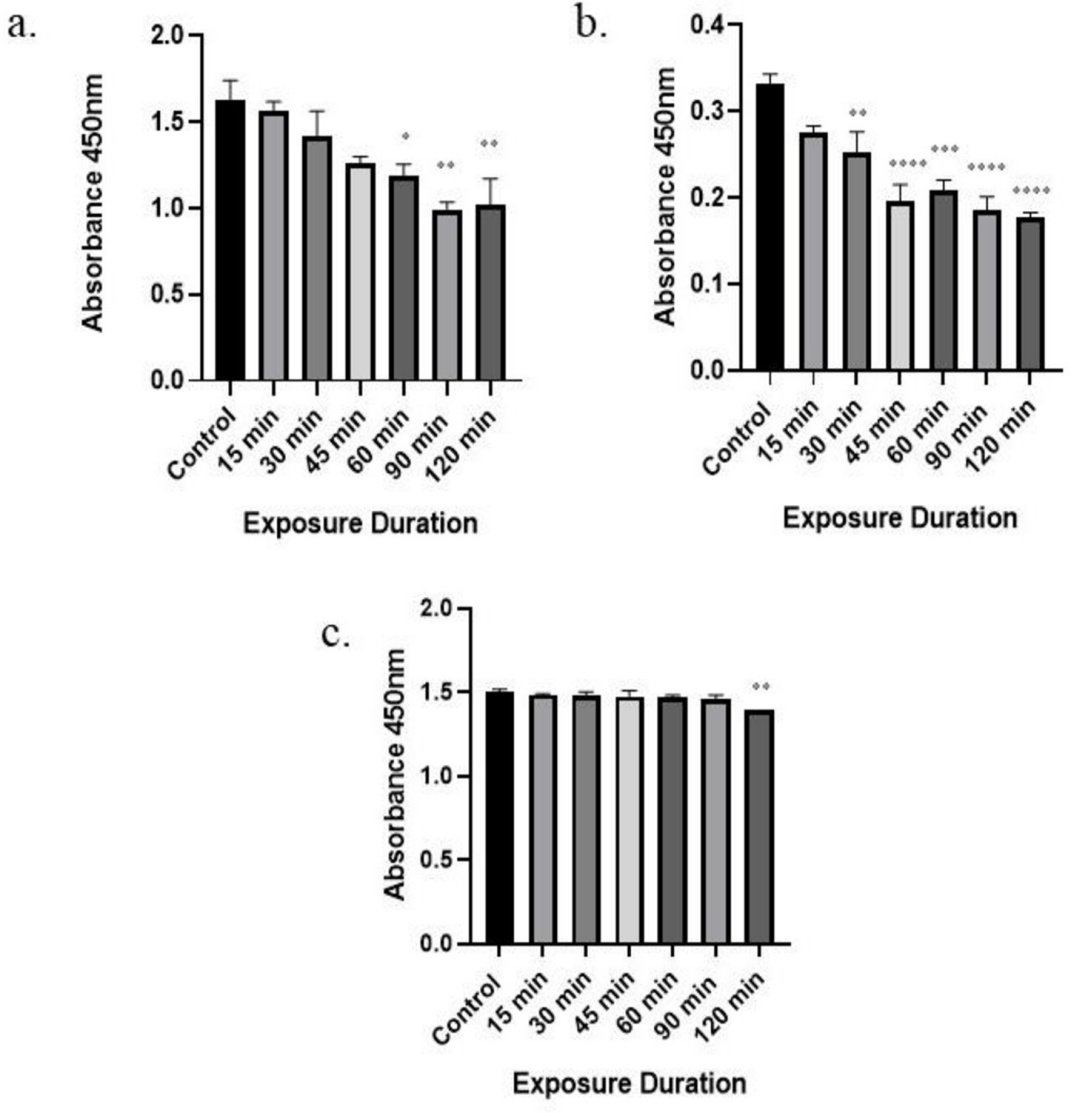
Time-dependent effect of radiation on proliferation of the Leydig cells. a) Mobile phone radiation, b) 2450 MHz, and c) 1800 MHz. Statistical significance with respect to the control group are shown as * (p < 0.05); ** (p< 0.01); *** (p < 0.001); **** (p < 0.0001) and value represents the mean of one independent experiment (n = 3for each group). Results are expressed as the mean ± standard error of the mean (SEM).

The MTT assay was used to evaluate the proliferation of cells, and the absorbance of formazan is used to express the proliferation of cells following exposure to radiation at frequencies of 1800 MHz and 2450 MHz provided by the exposure system and non-ionizing electromagnetic radiation from mobile phones. Statistical analysis through ANOVA showed that cell proliferation is dependent on irradiation time. The groups that differed significantly from the rest were separated using a multiple-comparison approach. After exposure to mobile phone radiation (Fig 2A), cell proliferation was found to be decreased with increasing duration of exposure. The significant decline in cell proliferation was noticed at 60min (p < 0.05), 90min (p< 0.01), and 120min (p< 0.01) of exposure in comparison to control group. Similarly, when cells exposed to 2450 MHz radiation from signal generator (Fig 2B), cell proliferation begin to decrease at 15 min exposure (p > 0.05) with significant drop from 30 min (p< 0.01) till 120 min of exposure (p < 0.0001) as compared to control group. Among various exposure (2450 MHz radiation) durations, 120 min of exposure appeared to be most effective as compared to other exposure durations.

On the other hand, when compared to control group, irradiation of 1800 MHz radiation from signal generator (Fig 2C) caused a significant decrease in cell proliferation only at 120 min (p < 0.05). However, there was no significant difference in the groups exposed for 15, 30, 45, 60, and 90 min (p > 0.05). The results shown in Fig 2 illustrates that there was significant decline in cell proliferation following exposure to RFR (1800 MHz, 2450 MHz, and mobile phone radiation) for varied intervals of time. Among various exposure durations, 120 min of exposure appeared to be most effective as compared to other exposure durations in all three exposure conditions (1800 MHz, 2450 MHz, and mobile phone radiation). Moreover, the induced trend in cell proliferation of two RFR exposure groups i.e. mobile phone radiation and 2450 MHz, appears somewhat similar.

### 3.3 Effect of RFR on testosterone levels

**Fig 3:**
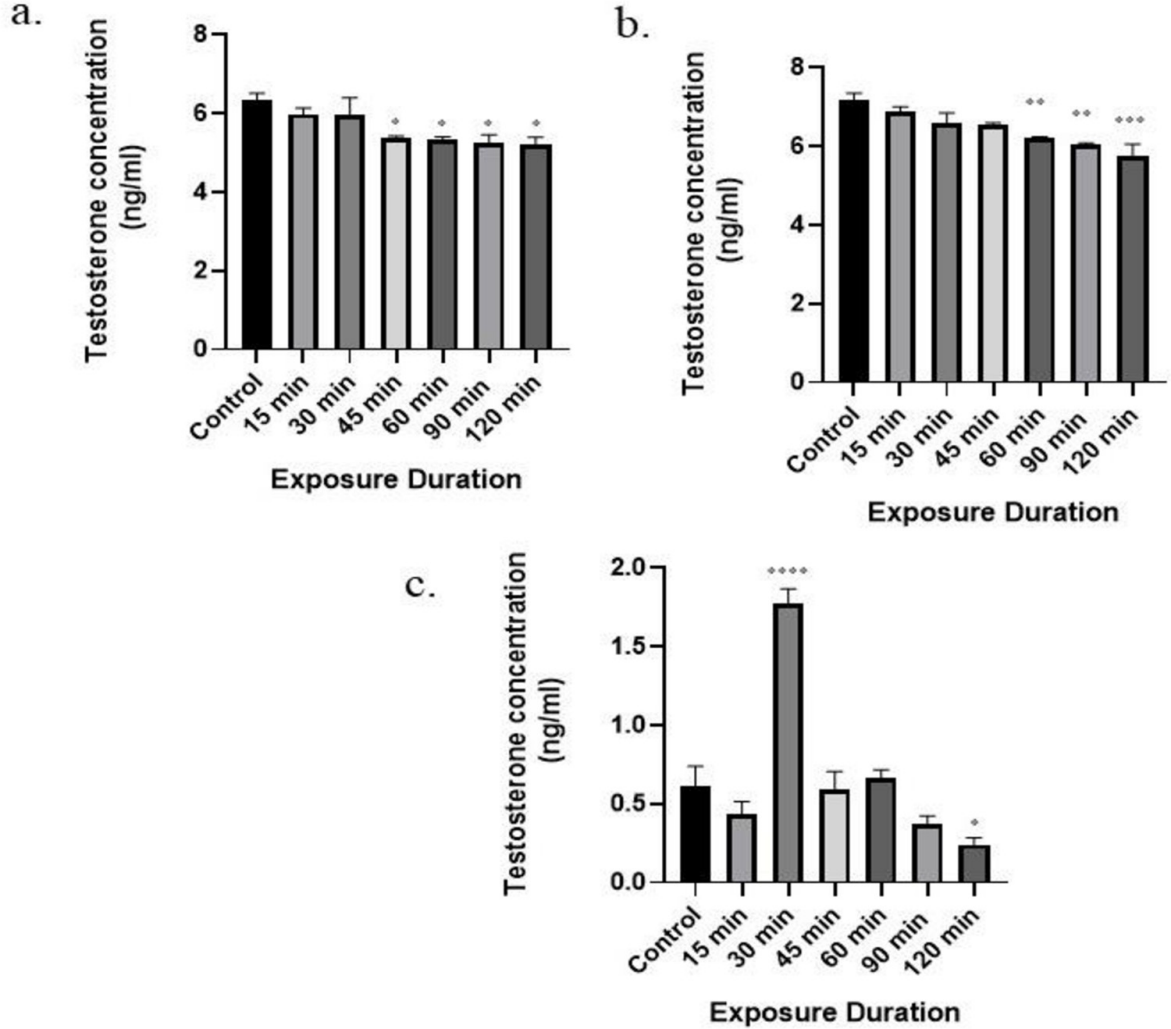
Time-dependent effect of radiation on levels of testosterone in the Leydig cell supernatants. a) Mobile phone radiations, b) 2450 MHz, and c) 1800 MHz. Statistical significance with respect to the control group are shown as * (p < 0.05); ** (p< 0.01); *** (p < 0.001); **** (p < 0.0001) and value represents the mean of one independent experiment (n = 3for each group). Results are expressed as the mean ± standard error of the mean (SEM).

Testosterone is considered an important parameter for Leydig cell function. Supernatant for testosterone levels detection were collected from all wells after *in vitro* exposure to 1800 MHz, 2450 MHz, and mobile phone radiation. The levels of testosterone were measured using the Cayman Enzyme Immunoassay kit *via* ELISA reader. It was observed that 1800 MHz, 2450 MHz, and mobile phone radiation exposure for varying periods dramatically altered the testosterone levels. When compared to control group, testosterone content in the supernatant of mobile phone radiation groups (45, 60, 90, and 120 min) was decreased significantly following exposure (p < 0.05; Fig 3A). Similarly, as compared to the control group, a significant decrease in the testosterone concentration was found in the supernatant of the 60, 90, and 120 min exposure groups of 2450 MHz radiation (p< 0.01,p< 0.01, p < 0.001 respectively; Fig 3B). However, irradiation to 1800 MHz radiation (Fig 3C) caused a significant increase in the testosterone levels at 30 min of exposure (p < 0.0001) but a subsequent decrease at 120 min (p < 0.05) of exposure duration in comparison to non-irradiated group. In all RFR exposure conditions (1800 MHz, 2450 MHz and mobile phone radiation), 120 min exposure found to be most effective among various exposure durations. Additionally, 2450 MHz and mobile phone radiation shows nice similarity in induced trend in testosterone levels.

### 3.4 Effect of RFR on intracellular ROS generation

The potential role of 1800 MHz, 2450 MHz, and mobile phone radiation in inducing oxidative stress was evaluated byfluorimetric assay using the oxidation sensitive fluorescent dye DCFH-DA to monitor ROS generation. DCFH-DA can passively diffuse into the cell through cell membrane, deacetylated by cellular esterases and in the presence of ROS, oxidized to fluorescent DCF. The measure of fluorescent intensity is used to determine the levels of ROS in the cells. A noticeable increase in the fluorescence intensity after mobile phone radiation exposure was observed at 60 min (p < 0.001), 90 min (p < 0.0001), and 120 min (p < 0.0001) in comparison to non-irradiated cells. Similarly, when compared to the non-irradiated group, a significant increase in the fluorescence intensity after 2450 MHz radiation exposure was observed at 30 min (p< 0.01), 45 min (p < 0.001), 60 min (p < 0.001), 90 min (p < 0.001), and 120 min (p < 0.0001). Whereas, fluorescence intensity demonstrated no significant (p > 0.05) effect in the levels of ROS at any of the time points after exposure to 1800 MHz radiation in comparison to non-irradiated cells.

**Fig 4:**
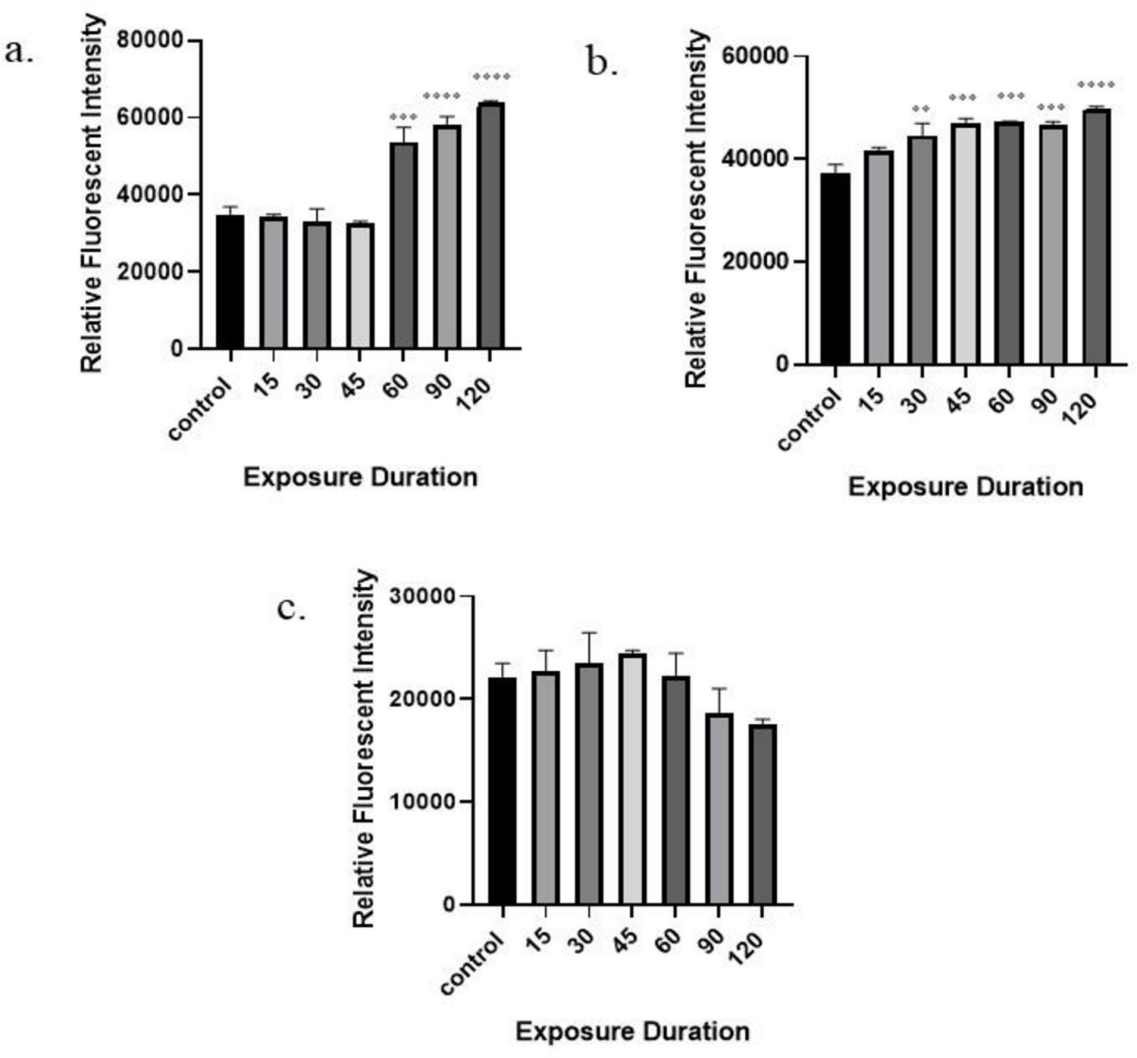
Time-dependent effect of radiation on intracellular ROS generation in Leydig cells. a) mobile phone radiations, b) 2450 MHz, and c) 1800 MHz. Statistical significance with respect to the control group are shown as * (p < 0.05); ** (p< 0.01); *** (p < 0.001); **** (p < 0.0001) and value represents the mean of one independent experiment (n = 3for each group). Results are expressed as the mean ± standard error of the mean (SEM).

Results of intracellular ROS generation are presented in Fig 4 and the data suggest that both 2450 MHz and mobile phone radiation exposure induces intracellular ROS generation whereas exposure to 1800 MHz radiation has no significant effect on ROS levels.

## 4. Discussion

Radiofrequency electromagnetic radiation is a significant factor in the impairment of reproductive functions [5, 6]. Leydig cells, the predominant cell type in the interstitium, perform a crucial regulatory role in male reproduction by secreting testosterone, and subsequently assist in spermatogenesis [13]. These cells are reported as most vulnerable cells to EMR exposure [24–29]. With such a concerning aspect and to get clarity, the current study aimed to investigate the difference in effects of different RFR exposure frequencies (1800 MHz, 2450 MHz, and mobile phone radiation) on the Leydig cell function over a range of exposure duration and intended to answer this research question with new perspectives. We have selected 1800 MHz and 2450 MHz RFR because most of the 3G, 4G mobile phones and Wi-Fi devices operate in this range. The duration of exposure to cellular phones is an important factor, but the exact duration differs from individual to individual. Hence, in this study, we chose the varying periods of exposure, i.e., 15, 30, 45, 60, 90, and 120 min, which are common cell-phone usage durations among individuals [30]. A broader range of exposure times, including both shorter and longer durations, would provide a more comprehensive understanding of the time-dependent effects of RFR on Leydig cell functions. We found that RFR exposure in TM-3 cells affects the Leydig cell functions in a time-dependent manner. When comparing different exposure times, 120 min seemed to be most effective in all exposure conditions because Leydig cell parameters were significantly altered at this exposure duration. The Leydig cell proliferation, testosterone production, and intracellular ROS generation were found to be altered significantly in 1800 MHz, 2450 MHz, and mobile phone radiation exposed groups. Previous studies have also reported a possible link between radiofrequency radiation exposure and male infertility [5, 15, 29, 31].

Leydig cells are responsible for the production of testosterone and subsequently supporting spermatogenesis to maintain male fertility [5, 13]. In our study, the testosterone levels in mobile phone exposure group were found to decrease at 45, 60, 90, and 120 min. Similarly, in case of 2450 MHz radiation exposure, the testosterone levels declined significantly at 60, 90, and 120 min. However, the testosterone levels were found to increase significantly at 30 min and subsequently decrease at 120 min of 1800 MHz radiation exposure. Several investigations are in agreement with our data, where testosterone levels were found to decrease with EMR exposure. The *in vivo* studies with mobile phone radiation exposure found that after 60 min or 120 min of exposure, the testosterone levels were decreased significantly while ROS production increased significantly in the exposed group compared to the control [10, 32, 33]. Similarly, in other studies, testosterone levels in the serum of rats were decreased when exposed to mobile phone radiations for 2 hr to 3hr for a period of 28 days and 3 months [32, 34]. The other study with an exposure frequency of 1950 MHz at SAR of 3 W/kg, also showed a significant decline in the testosterone levels and cell proliferation in TM3 cells after exposure of 24 hr for 5 days [29]. In addition, Chen et al. [35] showed that 1800 MHz exposure at a power density of 208 μW/cm^2^for 2 h/day for continuous 32 days induced a significant reduction in the serum testosterone levels in the exposed group as compared to the sham group. In contrast to our data, Forgács et al. [36] reported that exposure to 1800 MHz GSM-like microwave for 2hr/day with SAR of 0.018–0.023 W/kg, led to increased serum testosterone levels when compared to the control group which might be due to different study subjects (NMRI mice). However, this study also noticed that when exposure was given in culture to mice leydig cells, the rise in testosterone level was insignificant. This indicates that RFR’s impact on testosterone levels increase with exposure duration. The impaired testosterone production in EMR exposed groups could be due to the insufficient polarization of the cell as EMR might affect the polarization state of a cellular membrane [32].

Cell viability plays a significant role during cell culture and may have an impact on the quality of the results. The current investigation found no significant difference between non-irradiated and irradiated cells in terms of the viability of cultivated Leydig cells which is also supported by other authors [20, 37, 38]. Therefore, it is suggested that the changes seen in other parameters are not the result of a loss in cell viability.

It has been hypothesized that the electromagnetic field generated from radiofrequency electromagnetic radiation causes free radicals production, which results in oxidative stress [5–7, 20, 33]. Thus, we also evaluated intracellular ROS production in an effort to comprehend the probable processes behind the impact of RF-EMR. Our study demonstrates a significant increase in the ROS in TM-3 cells upon exposure to RFR of 2450 MHz and mobile phone radiation. In the mobile phone radiation and 2450 MHz radiation exposed groups, there is a considerable increase in the intracellular ROS levels at 60, 90, and 120 min of continuous exposure. Similarly, some authors have revealed that RF-EMF stimulates the development of ROS in the reproductive system [5, 6, 39, 40]. According to Lai and Singh [41], when rat brain cells were subjected to continuous and pulsed radiofrequency radiation at a frequency of 2450MHz with SAR of 1.2 W/kg for 2 hr each day, the free radical production in the body increased.

Supporting to our another study case (1800 MHz radiation), Lin et al. [29] reported insignificant differences in the ROS levels between the irradiated and the sham-exposed group, when Leydig cells were exposed to 1950 MHz radiation for 24 hr at SAR of 3 W/kg. Similarly, the other study reported that exposure to either single signal (837 MHz or 1950 MHz alone) or multiple signals (837 and 1950 MHz) at SAR of 4 W/kg for 2 hr, showed no statistically significant alterations in the ROS levels in human mammary epithelial cells [42]. Thus, our study concluded that the exposure situation (2450 MHz and Mobile phone radiation) induce intracellular ROS generation in TM3 cells, whereas, exposure to 1800 MHz radiation resulted in insignificant differences in intracellular ROS levels between the irradiated and the control group.

In order to maintain a normal fertile state, the proliferation of Leydig cells is crucial for the activation of spermatogenesis. In our investigation, exposure to RFR resulted in significant inhibition of Leydig cell proliferation as compared to control group. According to an *in vivo* study on reproductive parameters, exposure to non-ionizing radiation emitted from mobile phone has negative effects on the proliferation and differentiation of spermatogonia [43]. Ozgur et al. [44] noticed a substantial increase in cell proliferation after 1 hr of exposure to the 1800MHz RFR, whereas exposure to 900 and 1800 MHz (2 W/kg) RFR for 4 hr caused a significant reduction in the proliferation of Hep G2 cells. This suggests that the increase in exposure duration could decrease the cell proliferation rate, which is also observed in our study where 1800 MHz RFR substantially decreased Leydig cell prolifertion after 120 min of exposure. However, in another study, proliferation of HCT-116 and DLD-1cell lines was not inhibited by exposure to RF at 900, 1800 or 2100 MHz in a dose- or time-dependent manner [45]. These divergent results might be the consequence of type of research *(in vivo* or *in vitro* research), a variation in cell types, or different exposure conditions. When considering the RFR studies on same cell type *i*.*e*. Leydig cells, the cell proliferation in the irradiated group was inhibited significantly after exposure to 1950 MHz 3 W/kg radiation for 24 hr [5, 29]. According to Alghannam et al. [46], exposure of rats to mobile phone radiations for 60 min/day for 6 weeks resulted in decreased interstitial cell proliferation. In line with these facts our current findings demonstrate a significant decrease in cell proliferation in TM-3 cells upon mobile phone radiation exposure (60, 90, and 120 min), 2450 MHz radiation exposure (30, 45, 60, 90, 120 min), and 1800 MHz radiation exposure for 120 min, when compared to non- irradiated group.

RFR at the studied frequencies caused significant alteration in proliferation, testosterone production, and ROS generation in the examined cells (TM-3). In human cells (ASCs and Huh-7), 1750MHz LTE RF-EMF increases intracellular ROS, which slows down proliferation and accelerates senescence [47]. Hence, the increased ROS generation in our RFR exposed groups might be the reason for decline in proliferation rate of TM-3 cells. Numerous cellular processes, including autophagy and DNA damage, might be triggered by ROS. Elevated ROS can activate the cell apoptotic signaling cascade and excessive apoptosis may cause infertility in males [14]. Apoptosis in the Leydig cells can cause a decrease in the synthesis of testosterone [48] and it could be the reason why testosterone levels are declining following exposure to EMR. ROS increases oxidative stress and impairs the production of steroid hormones [49], and could be one of the reasons of decreased testosterone levels. The testicular ROS generation is responsible for possible detrimental effects on the reproductive parameters [24]. When extrapolated to men, the findings of this study and other studies of comparable kind must warn the scientific community that RFR may have long-term harmful consequences on reproductive health. Altogether, the findings suggest that RFR may affect the reproductive health and could cause infertility in males. Nevertheless, further research is required to determine the precise mechanism behind this process.

## 5. Conclusion

In conclusion, the present study demonstrates that the exposure to radiofrequency electromagnetic radiation can induce negative effects on the male reproductive system by impairing Leydig cell (TM3) functions, which includes decreased testosterone production, inhibited cell proliferation, and increased intracellular ROS generation in a time-dependent manner. At the same time, no effects were observed in terms of cell viability at any exposure duration and condition.

- The RFR effects appears to be exposure duration dependent as the severity of effects increase with increase in exposure duration. It is notable that RFR appears to be more damaging after 45 min to 60 min of continuous exposure. Besides, 120 minutes of exposure causes the maximum damage at all studied RFR conditions (1800 MHz, 2450 MHz, and mobile phone radiation).
- The 1800 MHz RFR from signal generator is observed to be less harmful than 2450 MHz RFR from signal generator and mobile phone. Besides, the effects of 2450 MHz RFR from signal generator and mobile phone are observed to have a pattern that is remarkably comparable.
- The RFR induced intracellular ROS levels could be the cause of suppression in TM3 cell proliferation, which might further lead to reduction in testosterone synthesis.
- These observations suggests that the changes caused are not a result of loss in cell viability of TM3 cells.
- Since very limited literature is accessible in this field for comparison and reference that could suggests relationship between Leydig cell functions and RFR, further research should be done on the other factors involved to better understand how radiofrequency radiation affects Leydig cell parameters and thereby male fertility. More research is also needed to comprehend the molecular mechanisms behind the reduced testosterone secretion caused by RF-EMR exposure to explore the apoptotic route as well as the steriodogenic pathway. Also, the study demands to validate these findings at in-vivo level, since, exposure to RFR is causing noticeable harmful changes in reproductive function that could induce infertility in males.

## Supporting information

S1 File Raw data of the graphs plotted. (XLSX)

## Abbreviations

RFR: radiofrequency radiation
ELF: extremely low frequency
EMR: electromagnetic radiation
ROS: reactive oxygen species
SAR: specific absorption rate
WHO: world health organization
Hz: hertz
MHz: Mega hertz
W/Kg: Watt/Kg
